# Differential analysis of brain functional network parameters in MHE patients

**DOI:** 10.1049/htl2.70004

**Published:** 2025-03-05

**Authors:** Li Song, Yiting Zhang, Xiaoyan Wang, Xucai Ji

**Affiliations:** ^1^ Radiology Department The Second Affiliated Hospital of Shandong First Medical University Tai'an China; ^2^ School of Radiology Shandong First Medical University & Shandong Academy of Medical Sciences Tai'an China; ^3^ College of Medical Information Engineering Shandong First Medical University &Shandong Academy of Medical Science Tai'an China; ^4^ College of Continuing Education Shandong First Medical University &Shandong Academy of Medical Science Tai'an China

**Keywords:** brain, medical image processing

## Abstract

Resting‐state functional magnetic resonance imaging, using blood‐oxygen‐level‐dependence signal data and graph theory, was employed to explore brain functional network parameter changes in 32 MHE patients and 21 healthy controls. The Gretna software package and spm8 are used to preprocess and process the data in matlab2012b to calculate the global efficiency (Eg), local efficiency (El), nodal degree (nodal De), nodal clustering coefficient (nodal Cp), nodal shortest path length (nodal Lp), and nodal betweenness (nodal Be) as brain functional network characteristic parameters. The BrainNet View soft is used to draw network maps and present surface‐based data. Within the sparsity range of the selected network, A double‐sample t‐test revealed significant differences about the characteristic parameters in the following brain regions: the Nodal Cp in AAL62, AAL26, AAL43, and AAL47; the De in AAL66, AAL68, AAL47, and AAL74; the nodal Lp in AAL28, the El in AAL62, AAL31, and AAL47; the Eg in AAL28, AAL32, and AAL51, and the nodal Be in AAL28, AAL32, AAL76, and AAL82. These changes in brain network nodes may signal early brain damage in MHE, helping to characterize MHE and predict mental decline in cirrhosis patients.

## Graphical abstract

Resting‐state functional magnetic resonance imaging with blood‐oxygen‐level‐dependence signal data and the graph method are used to explore changes in the characteristic parameters of the brain functional network in individuals with minimal hepatic encephalopathy compared with controls.

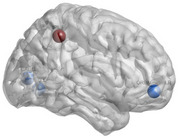

## 1 INTRODUCTION

Mild hepatic encephalopathy [1] (MHE) is a kind of hepatic encephalopathy. About 30–45% [2] of patients with cirrhosis will have secondary hepatic encephalopathy. Minimal hepatic encephalopathy (MHE) patients do not exhibit obvious clinical or neurological symptoms and can only be detected through neuropsychological methods to identify deficits in their neurocognitive functions [3].

Referring to previous neuroimaging studies on MHE [4], global efficiency and characteristic path length of brain networks can reflect the brain's functional integration capabilities. For local parameters of the brain network, patients show statistically significant changes in nodal degree centrality, nodal efficiency, and nodal shortest path length.

In view of this, this study focuses on the brain functional network of MHE patients. Based on the rs‐fMRI [5, 6, 7] imaging data, the brain functional network of MHE patients is constructed by using the graph theory method to explore the differences in the topological attribute parameters of the brain network between the MHE patients and the normal control group, in order to reveal the potential mechanism of the changes in the neural cognitive function [8] of the MHE patients.

## 2 MATERIALS AND METHODS

### 2.1 Data collection

The data used in this study were collected from 53 eligible participants, including 32 MHE patients who had been clinically diagnosed and 21 CNs. There were no significant differences in the demographic data between the two groups (*p* > 0.05). The diagnosis of MHE was mainly based on neuropsychological scores (PHES) for tests including the digital connection test (DCT) A/B and the digital symbol test (DST), among others. Two individuals with brain abnormalities in the experiment were diagnosed as having MHE [9]. The demographic data and clinical psychological test data from the two groups are listed in Table 1. We obtained blood‐oxygen‐level‐dependence (BOLD) signal data via rs‐fMRI scanning. During the scanning process, all participants were required to lie flat, relax, close their eyes, stay awake, and to not think about anything in particular or perform any tasks. The scanning parameters are listed in Table 2.

**TABLE 1:** Demographic and psychometric hepatic encephalopathy scores in patients with CN and MHE.

	CN (21)	MHE (32)	*P*‐value
Age (Y)	52.5 ± 6.3	53.6 ± 7.9	0.19
Sex (F/M)	5/16	8/24	0.59
Education (Y)	8.6 ± 2.2	7.2 ± 2.9	0.11
NCT‐A (s)	46.3 ± 15.3	76.6 ± 18.4	<0.001
NCT‐B (s)	112.5 ± 29.5	190.4 ± 42.0	<0.001
DST	44.5 ± 9.8	23.4 ± 4.3	<0.001

**TABLE 2:** Magnetic resonance scanning parameters.

Scanning parameters	Value
Repetition (TR)	2500 ms
Echo time (TE)	40 ms
Slice thickness (ST)	5 mm
Slice gap (SG)	1 mm
Storage matrix (SM)	64 × 64
Field of view	240 mm × 240 mm
Flip angle	90

### 2.2 Data processing

#### 2.2.1 Data preprocessing

Based on MATLAB 2012b platform, Gretna software package and spm8 were used to preprocess the collected rs‐fMRI data. The pretreatment steps mainly include:

1. Data format conversion: convert the original image format of all subjects to NIFTI format.
2. Removal of volumes: The fMRI data of the first 8 time points of each subject were removed.
3. Slice timing correction: Take the scanned middle layer as the reference layer.
4. Head movement correction (realignment): First calculate the head movement parameters (translation and rotation parameters in *X*, *Y*, and *Z* directions) of the scanned image, and remove the images with an average of more than 2.0 mm or rotation of more than 2.0 degrees among the head movement parameters.
5. Image registration.
6. Spatial normalization: The images after T1 joint segmentation were registered to the Montreal Institute of Neurology (MNI) template.
7. Spatial smoothing: The full width half height nucleus (FMH) was 6 × 6 × 6.
8. Linear detrends correction.
9. Filtering: bandwidth 0.01–0.08 Hz.
10. Remove covariates: The effects of head movement parameters, white matter signals and cerebrospinal fluid were removed.

#### 2.2.2 Functional network construction

The static functional network is constructed based on graph theory analysis. The processing steps are as follows:

1. The preprocessed brain imaging data were divided into 90 brain regions using the automated anatomical labelling (AAL) template. Each brain region represents a network node whose value is replaced by the arithmetic mean of the BOLD data of voxels in the corresponding brain region. The edge of the connected brain network is defined as the Pearson correlation coefficient of any two brain intervals, and a 90 × 90 correlation coefficient matrix of any two nodes is obtained. The correlation coefficient matrix is subjected to Fisher‐z transformation to obtain the correlation matrix.
2. A threshold value of 0.05≤ sparsity ≤0.50 and an interval of 0.01 is adopted to binarize the correlation matrix. If the correlation coefficient of any two brain regions in the brain network is greater than the predefined threshold value, it is converted to 1, indicating that the two nodes are connected; otherwise, it is converted to 0, indicating that the two nodes are not connected [10, 11, 12].
3. Calculate the graph theory indicators, and we use the graph theory analysis software Gretna, construct undirected weighted networks from the Pearson correlation coefficient matrix to calculate the Eg, El, nodal De, nodal Cp, nodal Lp and Nodal Be indicators. We applied a fixed thresholding method to create binary networks, set the threshold range of 0.1 to 0.5 with a step size of 0.01, and in SPM 8 we followed the default parameters for preprocessing.

## 3 RESULTS

**FIGURE 1:**
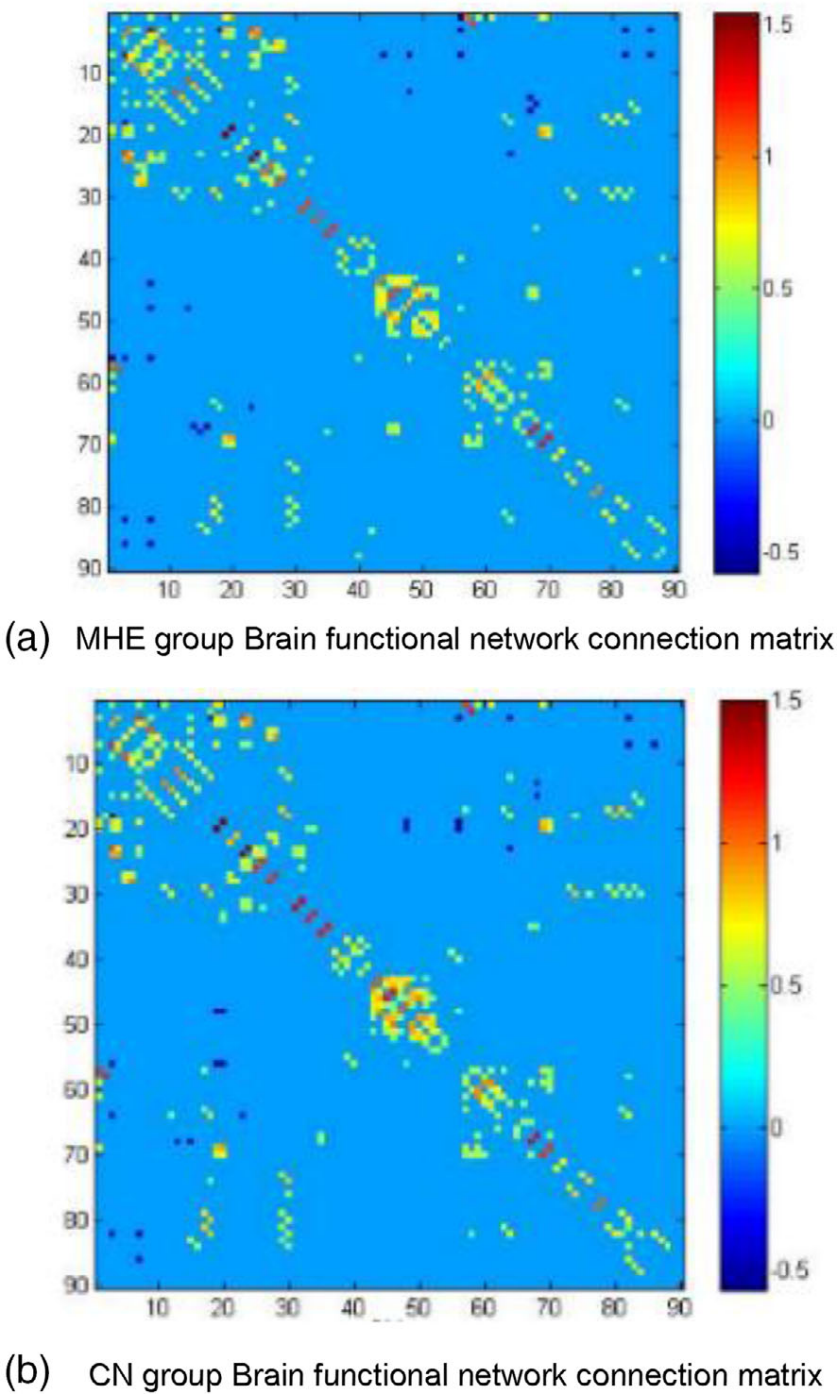
Brain functional network connection matrix (sparsity = 0.05).

**FIGURE 2:**
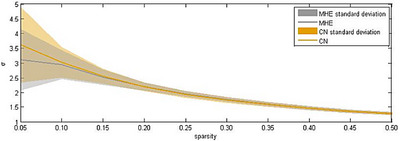
Changes in small world character parameter σ as a function of sparsity in the MHE and CN groups.

**FIGURE 3:**
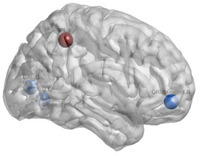
Brain regions with significant differences in nodal Cp. “Red” indicates an increase, “blue” a decrease.

**FIGURE 4:**
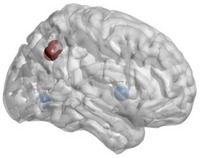
Brain regions with significant differences in De. “Red” indicates an increase, “blue” a decrease.

**FIGURE 5:**
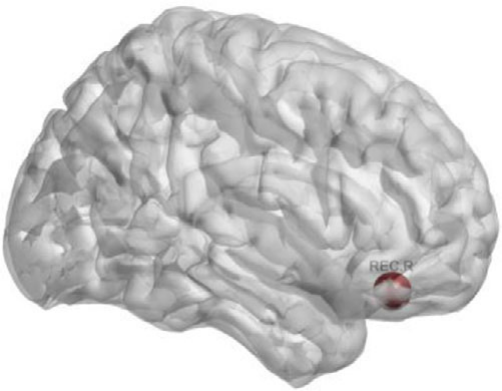
Brain regions with significant differences in nodal Lp.

**FIGURE 6:**
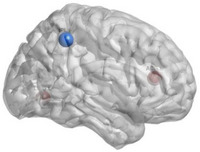
Brain regions with significant differences in *E*_l_.

**FIGURE 7:**
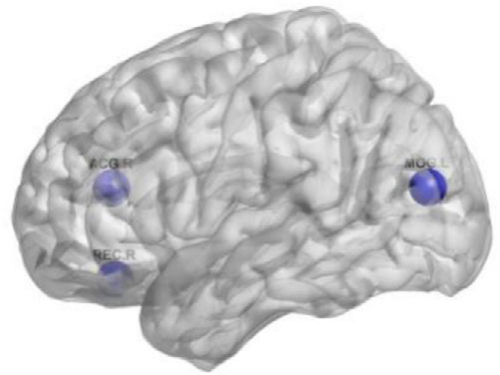
Brain regions with significant differences in *E*_g_.

**FIGURE 8:**
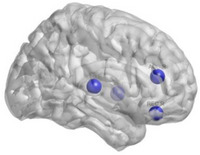
Brain regions with significant differences in nodal Be.

In this study, the characteristic parameters of the network nodes included *E*_g_, *E*_l_, nodal *D*_e_, nodal Cp, nodal Lp, nodal Be and so on. Figure 1 shows the 90 × 90 average brain network coefficient connective matrix. Figure 2 shows the variation in small‐world parameters as a function of the sparsity threshold. A double‐sample *t*‐test revealed increased nodal Cp in MHE patients in the AAL62 brain area (right inferior parietal, but supramarginal and angular gyrus), and decreased nodal Cp in the AAL26 (right middle frontal gyrus, orbital part), AAL43(left calcarine fissure and surrounding cortex), and AAL47 (left lingual gyrus) brain areas (Table 3, Figure 3). We found increased nodal *D*_e_ in the AAL66 (right angular gyrus) and AAL68 (right precuneus) brain areas, and decreased nodal *D*_e_ in the AAL47 and AAL74 (right lenticular nucleus, putamen) areas (Table 3, Figure 4). We found increased nodal Lp in the AAL28 (right gyrus rectus) brain area (Table 3, Figure 5), increased *E*_l_ in the AAL62 brain area, and decreased *E*_l_ in the AAL31 (left anterior cingulate and paracingulate gyrus) and AAL47 (Table 3, Figure 6) brain areas. Further, we found decreased *E*_g_ in the AAL28, AAL32 (Right anterior cingulate and paracingulate gyrus), and AAL51 (Left middle occipital gyrus) (Table 3, Figure 7). We found decreased nodal Be in the AAL28, AAL32, AAL76 (Right lenticular nucleus, Pallidum), and AAL82 (Right superior temporal gyrus) (Table 3, Figure 8) brain areas.

**TABLE 3:** Comparison of node attributes of brain functional networks between the MHE and CN groups.

Character parameters	Indexes	Brain region	Increase (↑) or decrease (↓)	*P*‐value
Nodal cluster coefficiency	AAL26	Right middle frontal gyrus, orbital part	↓	0.04201
	AAL43	Left calcarine fissure and surrounding cortex	↓	0.01474
	AAL47	Left lingual gyrus	↓	0.029049
	AAL62	Right inferior parietal, but supramarginal and angular gyri	↑	0.035806
Nodal degree	AAL47	Left lingual gyrus	↓	0.013747
	AAL66	Right angular gyrus	↑	0.007537
	AAL68	Right precuneus	↑	0.047244
	AAL74	Right lenticular nucleus, putamen	↓	0.009117
Nodal short path	AAL28	Right gyrus rectus	↑	0.035085
Nodal local efficiency	AAL31	Left anterior cingulate and paracingulate gyri	↓	0.045268
	AAL47	Left lingual gyrus	↓	0.018084
	AAL62	Right inferior parietal, but supramarginal and angular gyri	↑	0.043428
Nodal global efficiency	AAL28	Right gyrus rectus	↓	0.029361
	AAL32	Right anterior cingulate and paracingulate gyri	↓	0.044825
	AAL51	Left middle occipital gyrus	↓	0.036471
Nodal betweeness	AAL28	Right gyrus rectus	↓	0.045084
	AAL32	Right anterior cingulate and paracingulate gyri	↓	0.031075
	AAL76	Right lenticular nucleus, Pallidum	↓	0.029942
	AAL82	Right superior temporal gyrus	↓	0.021211

## 4 DISCUSSION

In this study, the characteristic parameters of the calculated brain functional network mainly include *E*_g_ (global efficiency), *E*_l_ (local efficiency), nodal *D*_e_ (nodal degree centrality), nodal Cp (clustering coefficient), nodal Lp (nodal shortest path length), and nodal Be (nodal betweenness centrality).

*E*_g_ indicates the average efficiency of communication between network nodes. For brain functional networks, higher global efficiency means that there can be more effective information exchange between brain regions. *E*_l_ describes the information transmission efficiency within the neighbourhood of a node when the connection between a node and its immediate neighbours fails. It reflects the network's fault tolerance and local information processing capability. Nodal *D*_e_ refers to the number of connections a node has within the network. Nodes with higher degrees in brain networks may correspond to key functional areas that are connected to many other regions. Nodal Cp indicates the tendency of nodes to cluster together in the network. For brain networks, this means that functionally related areas tend to form tight clusters. Nodal Lp refers to the number of the shortest paths between any two points in the network. Shorter shortest path lengths mean that the network has higher efficiency and can transmit information quickly. Nodal Be indicates the mediating role of a node within the network. Nodes with high nodal betweenness centrality are usually key channels for information transmission.

In this paper, we constructed the brain functional group network of MHE patients and CN group based on rs‐FMRI and graph theory, and compared the topological properties of the whole brain network between the two groups.

Our results show that under sparsity, the small world parameters *σ* of MHE group and CN group is greater than 1, it shows the network topology characteristics of “small world” (Figure 2). If the brain networks of MHE patients maintain or enhance small‐world properties, such as high clustering and short path lengths, this may indicate that the brain is able to overcome certain obstacles caused by the disease and maintain better cognitive functions. Conversely, if the network properties are impaired, such as decreased clustering or increased path lengths, this may nodal Be associated with cognitive decline, manifesting as symptoms such as reduced attention and memory impairment.

The abnormal topological structure of brain functional network mainly shows the changes of *E*_g_ and nodal Lp. Compared with the CN group, the MHE patients had decreased *E*_g_ and increased nodal Lp and *E*_g_ measure the global transmission capacity of the network [13]. A shorter nodal Lp is associated with a higher *E*_g_, and a faster rate of information transfer in the brain. The nodal Lp value of AAL28 in the MHE patients was higher than that in the CN group, while the *E*_g_ value of AAL28, AAL32, and AAL51 was lower than that in the CN group, indicating that the global transmission ability of these brain regions was decreased; The nodal Be is a very important index describing the importance of nodes in a network. This study found that the nodal Be of AAL28, AAL32, AAL76, and AAL82 in the MHE group was lower than that in the CN group; The nodal Cp, nodal *D*_e_, and *E*_l_ of nodes describe the qualities of local information transmission. The nodal Cp of AAL26, AAL43, and AAL47, nodal *D*_e_ of AAL32 and AAL47, and *E*_l_ of AAL31 and AAL43 in the MHE group were lower than those in the CN group. These data indicate that the ability of these brain regions to process local information has been weakened. We also found that the nodal Cp and *E*_l_ of AAL62 and nodal *D*_e_ of AAL66 and AAL68 in the MHE group were significantly higher than those in the CN group.

Consistent with previous studies [4, 14], It can be seen that the small‐world properties of brain networks, such as high clustering coefficients and short path lengths, are closely linked to cognitive function. Maintaining or enhancing these properties helps preserve good cognitive function, while their impairment can lead to cognitive decline. Therefore, studying the relationship between brain network metrics and cognitive impairment is crucial for understanding the cognitive function changes in MHE patients.

Our results also show that MHE patients have impaired topological properties of brain functional networks and have a stronger tendency to “random networks”. Our results also show that the brain functional network has strong flexibility and plasticity. The increase in the attributes of some nodes (except nodal Lp) may be a compensation mechanism, which enables the brain to maintain the ability of information integration and analysis to a certain extent.

## AUTHOR CONTRIBUTIONS

**Li Song**: Data curation; formal analysis; methodology; writing—original draft; writing—review and editing. **Yiting Zhang**: Writing—original draft; writing—review and editing. **Xiaoyan Wang**: Formal analysis; supervision; validation. **Xucai Ji**: Funding acquisition; supervision.

## CONFLICT OF INTEREST STATEMENT

The authors declare no conflicts of interest.

## Data Availability

Data derived from public domain resources.

## References

[1] Ferenci, P. , Lockwood, A. , Mullen, K. et al.: Hepatic encephalopathy— definition, nomenclature, diagnosis, and quantification: final report of the working party at the 11th World Congresses of Gastroenterology, Vienna, 1998. Hepatology 35(3), 716–721 (2002) 11870389 10.1053/jhep.2002.31250

[2] Lin, Y. , Fan, Y.P. : Neuropsychological test and investigation of mild hepatic encephalopathy in patients with liver cirrhosis. Chin. J. Hepatol. 19(1), 65–66 (2011) 10.3760/cma.j.issn.1007-3418.2011.01.020 21272466

[3] Jaipriya, D. , Sriharipriya, K.C. : A comparative analysis of masking empirical mode decomposition and a neural network with feed‐forward and back propagation along with masking empirical mode decomposition to improve the classification performance for a reliable brain‐computer interface. Front. Comput. Neurosci. 16, 1010770 (2022) 36405787 10.3389/fncom.2022.1010770 PMC9672820

[4] Chen, H.J. et al.: Disrupted topological organization of brain structural network associated with prior over hepatic encephalopathy incirrrhotic patients. Eur. Radiol. 28(1), 85–95 (2018) 28667481 10.1007/s00330-017-4887-8

[5] Chen, H.J. , Zhu, X.Q. , Jiao, Y. et al.: Abnormal baseline brain activity in low grade hepatic encephalopathy: aresting‐state fMRI study. J. Neurol. Sci. 318, 140–145 (2012) 22541365 10.1016/j.jns.2012.02.019

[6] Chen, H.J. , Zhu, X.Q. , Yang, M. et al.: Changes in the regional homogeneity of resting‐state brain activity in minimal hepatic encephalopathy. Neurosci. Lett. 507, 5–9 (2012) 22178142 10.1016/j.neulet.2011.11.033

[7] Ling, N. , Qi, R.F. , Zhang, L.J. et al.: Altered regional homoseneity in the development of minimal hepatic encephalopathy: a resting state functional MRI study. PLoS One 7(7), e42016 (2012) 22848692 10.1371/journal.pone.0042016 PMC3404989

[8] Jaipriya, D. , Sriharipriya, K.C. : Brain computer interface‐based signal processing techniques for feature extraction and classification of motor imagery using EEG: a literature review. Biomed. Mater. Dev. 2(2), 601–613 (2024)

[9] Chinese Society of Hepatology : Guidelines on the management of hepatic encephalopathy in cirrhosis. Infect. Dis. Inf. 31(5), 403–420 (2018)

[10] Tian, L. et al.: Hemisphere‐ and gender‐related differences in small‐world brain networks: a resting‐state functional MRI study. Neuroimage 54(1), 191–202 (2011) 20688177 10.1016/j.neuroimage.2010.07.066

[11] Luo, C.Y. et al.: Functional connectome assessed using graph theory in drug‐naive Parkinson's disease. Neurology 262(6), 1557–1567 (2015) 10.1007/s00415-015-7750-3 25929663

[12] He, Y. et al.: Impaired small‐world efficiency in structural cortical networks in multiple sclerosis associated with white matter lesion load. Brain 132(12), 3366–3379 (2009) 19439423 10.1093/brain/awp089 PMC2792366

[13] Wang, X.Y. , Liu, S.S. , Wang, P.C. et al.: Small world characteristics of brain functional magnetic resonance network in type 2 diabetic encephalopathy patients. Chin. J. Med. Phys. 33(5), 501–504 (2016)

[14] Lv, X.F. et al.: Anomalous gray matter structural networks in patients with hepatitis B virus‐related cirrhosis without overt hepatic encephalopathy. PLoS One 10(3), e0119339 (2015) 25786256 10.1371/journal.pone.0119339 PMC4364769

